# Immune monitoring of SARS-CoV-2-specific T cell and B cell responses in patients with multiple sclerosis treated with ocrelizumab

**DOI:** 10.3389/fimmu.2023.1254128

**Published:** 2023-09-20

**Authors:** Elina Groß-Albenhausen, Alicia Weier, Markus Velten, Thorsten Heider, Rittika Chunder, Stefanie Kuerten

**Affiliations:** ^1^Institute of Neuroanatomy, Faculty of Medicine, University of Bonn and University Hospital Bonn, Bonn, Germany; ^2^Department of Anesthesiology and Intensive Care Medicine, University Medical Center Bonn, Bonn, Germany; ^3^Clinic for Neurology, Klinikum St. Marien Amberg, Amberg, Germany

**Keywords:** antibodies, B cells, CD4^+^ T cells, CD8^+^ T cells, mRNA vaccine, multiple sclerosis, ocrelizumab, SARS-CoV-2

## Abstract

**Introduction:**

Since the development of the coronavirus disease (COVID-19) pandemic caused by severe acute respiratory syndrome coronavirus type 2 (SARS-CoV-2), there has been significant interest in determining the effectiveness of SARS-CoV-2 vaccines in patients under immunomodulatory or immunosuppressive therapies. The aim of this study was to evaluate the impact of ocrelizumab, a monoclonal anti-CD20 antibody, on SARS-CoV-2-specific T cell and B cell responses in patients with relapsing-remitting multiple sclerosis (RRMS).

**Methods:**

To this end, peripheral blood mononuclear cells (PBMCs) were isolated from *n* = 23 patients with RRMS. Of these patients, *n* = 17 were tested before (time point t_0_) and one month after (time point t_1_) their first dose of ocrelizumab. In addition, we studied *n* = 9 RRMS patients that got infected with SARS-CoV-2 over the course of ocrelizumab therapy (time point t_2_). PBMCs were also isolated from *n* = 19 age- and gender-matched healthy controls (HCs) after vaccination or infection with SARS-CoV-2, respectively. Interferon-γ (IFN-γ)/interleukin-2 (IL-2) and granzyme B (GzB)/perforin (PFN) double-color enzyme-linked immunospot (ELISPOT) assays or single-color ELISPOT assays were performed to measure SARS-CoV-2 antigen-specific T cell and B cell responses. Anti-viral antibody titers were quantified in the serum by chemiluminescence immunoassay.

**Results:**

Our data indicate a significant difference in the SARS-CoV-2 specific IFN-γ (*P* = 0.0119) and PFN (*P* = 0.0005) secreting T cell compartment in the MS cohort at t_0_ compared to HCs. Following the first dose of ocrelizumab treatment, a significant decrease in the number of SARS-CoV-2 spike protein-specific B cells was observed (*P* = 0.0012). Infection with SARS-CoV-2 in MS patients under ocrelizumab therapy did not significantly alter their existing immune response against the virus. Kaplan-Meier survival analysis suggested that the spike S1 protein-specific immunoglobulin (Ig)G response might be a key parameter for predicting the probability of (re)infection with SARS-CoV-2.

**Discussion:**

Our results call for a critical discussion regarding appropriate vaccination intervals and potential biomarkers for the prediction of (re)infection with SARS-CoV-2 in patients with MS receiving ocrelizumab.

**Unique identifier:**

DRKS00029110; URL: http://apps.who.int/trialsearch/.

## 1 Introduction

Multiple sclerosis (MS) is a chronic autoimmune demyelinating disorder of the central nervous system (CNS) (1). It is the most frequent neurological disorder in young adults leading to irreversible deficits and premature retirement (1, 2). The immunopathology of MS is complex and incompletely understood but is said to involve cellular and molecular components of both the innate and adaptive immune system (3). While MS was long thought to be T cell-mediated (4), mounting evidence emphasizes the importance of B cells in mediating MS pathology by antibody-dependent and -independent mechanisms (5, 6). For instance, evidence of involvement of B cells in MS comes from neuropathological analysis of lesions from patients (7–9). Furthermore, the beneficial effects of anti-CD20 therapies underline the pathogenic involvement of B cells in MS (6).

A monoclonal humanized anti-CD20 antibody which is an approved treatment option for both relapsing-remitting MS (RRMS) and primary progressive MS (PPMS) is ocrelizumab, which has been shown to be highly effective in depleting circulating CD20^+^ B cells (10, 11). In pilot studies for the drug, ocrelizumab was compared to interferon-β1a therapy in RRMS patients (OPERA trial) (10) or placebo in PPMS patients (ORATORIO trial) (11). Both studies demonstrated a significantly reduced relapse rate and risk of disability progression, as well as a significantly lower number of magnetic resonance imaging (MRI) lesions in patients treated with ocrelizumab (10, 11).

However, given the immunosuppressive mode of action of anti-CD20 depleting antibodies, treatment with ocrelizumab also introduces an increased risk of infection as well as reduced vaccine effectiveness in patients with MS (12–14). With severe acute respiratory syndrome coronavirus type 2 (SARS-CoV-2) inducing a global pandemic, there has been renewed interest in establishing the effectiveness of the different SARS-CoV-2 vaccines in patients receiving immunomodulatory or immunosuppressive therapies.

There are several ways of measuring the effect of vaccination on the cellular and humoral compartments of the immune system. Some of the methods include intracellular cytokine staining and flow cytometry (15, 16), the enzyme-linked immunospot technique (ELISPOT) (17) as well as multiplex assays based on fluorescent beads or chemiluminescence (18). One of the most commonly used methods is ELISPOT that was first described by Czerkinsky et al. in 1983 (19) and relies on the measurement of antigen-specific immune responses on a single-cell level. ELISPOT is typically performed on peripheral blood mononuclear cells (PBMCs) and used to detect either antigen-specific cytokine secretion by T cells or antibody release by B cells (20–24). In particular, T cell ELISPOT has previously been used for evaluating antigen-specific T cell responses following viral infections and vaccinations, including in the case of coronavirus disease (COVID)-19 (25–27).

Here, we used ELISPOT as a method to evaluate SARS-CoV-2-specific T cell and B cell responses in RRMS patients in the context of ocrelizumab therapy. To this end, T cell and B cell ELISPOT assays were performed before and after the first dose of ocrelizumab, as well as one to three months after infection with SARS-CoV-2 in patients with RRMS. Results were compared to healthy controls (HCs) that were included either after vaccination against or infection with SARS-CoV-2. So far, only one study directly compared SARS-CoV-2-specific B cell responses and antibody titers before and after the initial dose of ocrelizumab in a small cohort (*n* = 4) of MS patients that had been vaccinated against the virus (28). The authors concluded that a recall immunity to SARS-CoV-2 present during ocrelizumab therapy could be successfully boosted by a third dose of the vaccine. Their data also suggested that terminally differentiated plasma cells were able to maintain a humoral immune response despite anti-CD20 therapy. Our results extend these findings to provide further information on SARS-CoV-2 specific T cell and B cell responses and their presence or persistence at different time points before and after administration of ocrelizumab, respectively.

To our knowledge, this is the first study to include both granzyme B (GzB) and perforin (PFN) as signature cytokines for cytotoxic T cells (29) using ELISPOT, allowing a more detailed evaluation of the impact of ocrelizumab on the adaptive immune compartment in the context of SARS-CoV-2 infection.

## 2 Methods

### 2.1 Study subjects

#### 2.1.1 MS cohort

All MS patients (*n* = 23; *n* = 18 females and *n* = 5 males) were recruited from the Klinikum St. Marien Amberg. They were over 18 years of age (mean age of 35.7 ± 8.4 years) and fulfilled the McDonald criteria for the diagnosis of RRMS (mean *Expanded Status Disability Scale* (EDSS) of 3.3 ± 1.2 and mean disease duration of 6.5 ± 7.8 years). All patients had been vaccinated between 2-5 times (*n* = 3 were vaccinated twice; *n* = 14 were vaccinated thrice; *n* = 5 and *n* = 1 were vaccinated four or five times, respectively) with either BNT162b2 (Comirnaty^®^) (Pfizer Inc., and BioNTech) or mRNA-1273 (Moderna, Inc.) 4.77 ± 3.22 months before being included in the study, with the exception of *n* = 1 patient who had received Novavax (NVX-CoV2373) 7.5 months prior to inclusion into the study. Most patients were under disease-modifying therapy (DMT) prior to their recruitment into the study. Patients that were not treated with ocrelizumab or who decided not to get vaccinated were excluded from the study. General information and clinical details of the MS cohort has been summarized in **Table 1**.

**Table 1:** Demographics of the MS patient cohort.

ID	Age	Sex	EDSS at t_0_	Age at diagnosis	Disease duration (Y)	DMT before ocrelizumab^b^	Number of SARS-CoV-2 vaccinations	Vaccine type; time since vaccination (t_0_)	Time since SARS-CoV-2 infection (t_2_)
MS-1	43	M	2	42	1	Glatiramer acetate	3	BioNTech; 3.5 months	/
MS-2	31	F	3	30	1	Plasmapheresis	3	BioNTech; 3 months	2 months
MS-3	23	M	4	22	1	Plasmapheresis	3	BioNTech; 3 months	/
MS-4	32	F	3.5	32	0	No treatment	3	Moderna; 5 months	N/A^a^
MS-5	48	F	4	18	30	Siponimod	3	BioNTech; 7 months	/
MS-6	25	F	2	25	0	No treatment	3	BioNTech; 5.5 months	2 months
MS-7	32	M	3	27	5	Dimethyl fumarate	4	BioNTech; 3.5 months	/
MS-8	30	F	2	21	9	Fingolimod	3	BioNTech; 4 months	/
MS-9	38	M	4	31	7	Fingolimod	2	BioNTech; 4 months	3 months
MS-10	41	M	3.5	39	2	Glatiramer acetate	3	BioNTech; 0.5 months	1 month
MS-11	49	F	2	46	3	No treatment	5	BioNTech; 1.5 months	2 months
MS-12	32	F	2.5	32	0	Plasmapheresis	3	BioNTech; 2.5 months	3 months
MS-13	39	F	3	33	6	Natalizumab	3	Moderna; 5.5 months	3 months
MS-14	43	F	3	27	16	Natalizumab	3	BioNTech; 5 months	1 month
MS-15	33	F	4	22	11	Fingolimod	3	BioNTech; 2.5 months	/
MS-16	31	F	2	29	2	Ponesimod	4	BioNTech; 2 months	/
MS-17	22	F	3.5	19	3	IFN-β	4	BioNTech; 4 months	/
MS-18	47	F	6.5	42	5	No treatment	3	BioNTech; 4 months	/
MS-19	34	F	4.5	18	16	Fingolimod	3	BioNTech; 9 months	2 months
MS-20	23	F	2	22	1	No treatment	2	BioNTech; 15 months	/
MS-21	36	F	2.5	34	2	Dimethyl fumarate	4	Moderna; 6 months	/
MS-22	41	F	2.5	34	7	No treatment	2	Novavax; 7.5 months	/
MS-23	49	F	5	31	18	Teriflunomide	4	BioNTech; 10 months	/

a This patient was infected with SARS-CoV-2 but not included in the blood/serum analysis due to pregnancy.

b Dimethyl fumarate = methyl ester of fumaric acid; fingolimod/ponesimod/siponimod = sphingosine-1-phosphate receptor modulator; glatiramer acetate = random polymer composed of four amino acids; IFN-β = cytokine of the interferon family; natalizumab = humanized monoclonal antibody against the cell adhesion molecule α4-integrin; teriflunomide = inhibitor of pyrimidine de novo synthesis by blocking the enzyme dihydroorotate dehydrogenase.

DMT, disease-modifying therapy; EDSS, expanded disability status scale; f, female; m, male; Y, years.

For isolation of PBMCs, 36 mL of heparinized peripheral blood was collected immediately before the first treatment cycle with ocrelizumab (baseline, time point t_0_) and four weeks after (time point t_1_) (*n* = 21 patients). An additional time point (t_2_) was defined as 2.1 ± 0.8 months after SARS-CoV-2 infection in patients (*n* = 9) that were already under ocrelizumab therapy. For the analysis of serum antibody titers, an additional volume of 4.7 mL of peripheral blood was collected in standard serum separator tubes at the designated time points. The research protocol was approved by the Ethics Committee of the Medical Faculty of the University of Bonn, Germany (file 387/21). Written informed consent was obtained from all participants.

#### 2.1.2 Healthy controls

*n* = 19 HCs (mean age 38.2 years ± 11.7; *n* = 11 females and *n* = 8 males) were either recruited from the Department of Anesthesiology and Intensive Care Medicine or the Institute of Neuroanatomy, University Hospital Bonn. Exclusion criteria were an age under 18, any underlying autoimmune or neurological disorder or treatment with immunomodulatory drugs (**Table 2**). All HCs had been vaccinated against SARS-CoV-2 at least twice either with BNT162b2 (Comirnaty^®^) (Pfizer Inc., and BioNTech) or mRNA-1273 (Moderna, Inc.) (*n* = 9 were vaccinated thrice; *n* = 7 were vaccinated four times and *n* = 3 were vaccinated five times). 40 mL of heparinized peripheral blood and 4.7 mL of peripheral blood in standard serum separator tubes was collected 1.73 ± 0.9 months after the last vaccination, or 1.5 ± 0.76 months after SARS-CoV-2 infection, respectively. The study was approved by the Ethics Committee of the Medical Faculty of the University of Bonn, Germany (file 022/22). Written informed consent was obtained from all participants.

**Table 2:** Demographics of the healthy control cohort.

ID	Age	Sex	Number of SARS-CoV-2 vaccinations	Reason for inclusion into the study	Time since vaccination or infection
HC-1	22	F	4	Vaccination (BioNTech)	2 months
HC-2	33	M	3	Infection	1 month
HC-3	45	M	3	Infection	1 month
HC-4	22	F	3	Infection	1 month
HC-5	22	F	4	Vaccination (BioNTech)	1 month
HC-6	22	F	4	Vaccination (BioNTech)	2 months
HC-7	47	F	4	Vaccination (BioNTech)	1 month
HC-8	46	M	5	Vaccination (BioNTech)	1 month
HC-9	44	M	3	Infection	2 months
HC-10	38	F	4	Vaccination (Moderna)	3 months
HC-11	31	F	5	Vaccination (Moderna)	3 months
HC-12	37	M	4	Vaccination (BioNTech)	1 month
HC-13	52	M	5	Vaccination (BioNTech)	1 month
HC-14	43	M	3	Vaccination (Moderna)	3 months
HC-15	41	F	4	Vaccination (BioNTech)	1 month
HC-17	39	F	3	Infection	2 months
HC-18	39	F	3	Infection	1 month
HC-19	68	F	3	Infection	1 month
HC-20	35	M	3	Infection	3 months

HC, healthy control.

### 2.2 Isolation of PBMCs

Peripheral venous blood (36 – 40 mL from every individual) was collected in heparinized tubes and mixed with phosphate-buffered saline (PBS) at a ratio of 1:1. PBMCs were isolated by Ficoll^®^ (Cytiva, #17144003) density gradient centrifugation and resuspended in Roswell Park Memorial Institute (RPMI)-1640 medium (Gibco^™^, #11875093). The cells were washed thrice with RPMI-1640, before being counted using acridine orange (Sigma Aldrich, #A9231). The concentration was adjusted to 3 x 10^6^ cells/mL and cells were resuspended either in RPMI-1640 supplemented with 1% penicillin/streptomycin (PenStrep) (Sigma, #P4333) and 10% fetal bovine serum (FBS) (Invitrogen, #10082147), or in CTL-Test^™^ medium (CTL, #CTLT-010).

### 2.3 ELISPOT assays

#### 2.3.1 T cell ELISPOT

For quantification of the T cell response, double-color interferon-γ/interleukin-2 (IFN-γ/IL-2) and granzyme B (GzB)/perforin (PFN) T cell ELISPOT assays were performed using ImmunoSpot^®^ kits (CTL, #SKU: hIFNgIL2-2M and #SKU: hGzBPFN-2M) following the manufacturer’s instructions. Briefly, polyvinylidene difluoride (PVDF) membrane plates were coated with capture antibodies against IFN-γ + IL-2 or GzB + PFN and incubated at 4°C overnight. Subsequently, the plates were washed with sterile PBS and the following antigens were added: (1) PepTivator^®^ SARS-CoV-2 Select Peptide Pool (Miltenyi Biotec, #130-127-309) used at a concentration of 15 nmol/mL to assess the SARS-CoV-2-specific T cell response; (2) CERI MHC class I peptide pool (containing antigens of cytomegalovirus (CMV), Epstein-Barr virus (EBV), human respiratory syncytial virus (hRSV), and influenza) (CTL, #CTL-CERI-300) used at a concentration of 1 µg/mL as the positive control for CD8^+^ T cells and (3) L-phytohaemagglutinin (PHA) (Sigma-Aldrich, #L2769-2MG) at a concentration of 5 µg/mL used as the positive control for the detection of CD4^+^ T cells.

SARS-CoV-2 antigen was coated in triplicates, while the control antigens were coated in singlicates. In *n* = 4 cases, ThermoFisher SARS-CoV-2 S1 protein aa11-682 (ThermoFisher, #RP-87679) was used instead of PepTivator^®^ at baseline (t_0_). Anti-CD28 antibody (CTL, #SKU: hIFNgIL2-2M) was added to the antigens at a concentration of 0.1 µg/mL for IFN-γ/IL-2 assays according to manufacturer’s instructions. Wells that contained CTL-Test^™^ cell culture medium only were used as the negative control (also coated in triplicates).

PBMCs were resuspended in CTL-Test^™^ medium and plated at a concentration of 300,000 cells/well. The plates were incubated at 37°C and 7% CO_2_ either for 23-25 h in the case of IFN-γ/IL-2 assays, or for 47-49 h in the case of GzB/PFN assays. Subsequently, they were washed with PBS and PBS-Tween (PBS-T; 0.05% Tween-20) and incubated either with biotinylated anti-IL-2 or anti-PFN, fluorescein isothiocyanate (FITC)-labeled anti-IFN-γ or HRP-coupled anti-GzB detection antibodies diluted to a concentration of 0.4% (IFN-γ) or 0.1% (IL-2, GzB, PFN) for 2 h at room temperature. Streptavidin conjugated to alkaline phosphatase (AP) was added to all plates. For IFN-γ/IL-2 assays, FITC coupled to horseradish peroxidase (FITC-HRP) was added in addition to streptavidin-conjugated AP. After an incubation time of 1 h at room temperature, the plates were washed with PBS-T and distilled water, and the blue developer solution was added for 20 min at room temperature. The plates were first rinsed with tap water, washed with distilled water and then the red developer solution was added for 20 min at room temperature. Both developer solutions were included in the ImmunoSpot^®^ kits (CTL, #SKU: hIFNgIL2-2M and #SKU: hGzBPFN-2M). Plates were finally rinsed with tap water and left to dry overnight. Analysis was performed using an ImmunoSpot^™^ Series 6 Universal Analyzer (CTL, ImmunoSpot^®^ 6.0.0.0 Professional DC). Spot counts were defined as too numerous to count (TNTC), when they were > 500/well for IFN-γ (30), > 400/well for IL-2 (31), > 700 for GzB/well and > 1000/well for PFN (the latter two values were defined based on our own experience).

#### 2.3.2 B cell ELISPOT

For the quantification of the B cell response, single-color ELISPOT assays were performed according to the protocol as previously described by our group (32). Briefly, after counting the cells, PBMCs were resuspended in RPMI-1640 supplemented with 10% FBS and 1% PenStrep. Cells were adjusted to a concentration of 3 x 10^6^ cells/mL before 1 µM β-mercaptoethanol (Sigma, #M7522), 5 µg/mL toll-like receptor-7 and -8 agonist R-848 (Enzo, #ALX-420-038-M005) and 15 ng/mL IL-2 (PeproTech, #200-02) were added for polyclonal B cell stimulation. Cells were then incubated in cell culture flasks (Greiner, #690175) at 37°C and 7% CO_2_ for 6 days.

On day 5, 96-well ELISPOT plates (Merck, #MSIPN4W50) were coated as follows: (a) 10% FBS in sterile PBS (as a negative control); (b) mouse anti-human IgG-1k (SouthernBiontech; #SBA-9230-01) (as a positive control); (c) SARS-CoV-2 spike S1 + S2 proteins (s-ECD region, amino acids 14-213; Thermo Fisher, #RP-87668) or, (d) SARS-CoV-2 nucleocapsid (NC) recombinant protein (Thermo Fisher; #RP-87665). The negative and positive controls were coated in triplicates and SARS-CoV-2 antigens in duplicates. On day 6, the plates were blocked with 10% FBS in sterile PBS at room temperature for 2 h, washed with sterile PBS and the polyclonally stimulated B cells were added at a concentration of 1 x 10^6^ cells/well. Plates were incubated at 37°C and 7% CO_2_ for 24-26 h. Biotinylated anti-human IgG secondary antibody (Mabtech, #MAB-3850-6-250) was then diluted to a concentration of 0.1 µg/mL in 1% bovine serum albumin (BSA) in PBS, added to the plates and incubated at 4°C overnight. Subsequently, streptavidin conjugated to AP (Vector Laboratories, #SA-5100) was added and incubated at room temperature for 2 h. Finally, plates were developed for 20 min using the Vector^®^ Blue AP Kit III (Vector Laboratories, #SK-5300). The plates were left to dry overnight before being analyzed with the ImmunoSpot^®^ Series 6 Universal Analyzer (CTL, ImmunoSpot^®^ 6.0.0.0 Professional DC).

### 2.4 Chemiluminescence immunoassay assay

For the evaluation of the humoral immune response to SARS-CoV-2, serum samples were sent to SYNLAB Weiden, Germany. SARS-CoV-2 spike S1 protein-specific antibody titers were quantified using a chemiluminescence microparticle immunoassay (SARS-CoV-2 IgG II Quant; Abott). Results were expressed as BAU/mL, where BAU refers to binding antibody units. The cut-off as defined by SYNLAB was < 7.10. Values in the range of 7.1 to 71.9 were considered as weakly positive, while values above 72 were considered as positive.

### 2.5 Statistical analysis

Power calculation was done in consultation with our colleagues from the Institute for Medical Biometry, Informatics and Epidemiology (IMBIE), University of Bonn. The software G*Power (version 3.1) was used for power calculation with the following set of parameters: two-tailed Mann-Whitney *U* test with a significance level of *P* = 0.05, type II error (β) = 0.8, effect size = 0.8; allocation ratio 1:1. GraphPad Prism version 9 (GraphPad Software, Inc.) was used for all statistical analyses. A Shapiro-Wilk normality test was used to determine normal distribution of the data sets and QQ plots were generated for verification. A Mann-Whitney *U* test was used with a significance level of 5% to assess statistical significance between the different groups and conditions. Kaplan-Meier survival analysis was performed to determine the probability of (re)infection with SARS-CoV-2 based on the number of SARS-CoV-2-specific T cells, B cells or serum antibody titers in both the MS and HC cohort. Additionally, receiver operating characteristic (ROC) analysis was performed to determine test performance (confidence intervals for sensitivity, specificity and accuracy were calculated using the exact Clopper-Pearson method).

## 3 Results

### 3.1 MS patients under immunomodulatory therapy differ from healthy donors in their antigen-specific immune response to SARS-CoV-2 and other viral antigens

At first, we set out to compare the SARS-CoV-2-specific immune response in the MS cohort at baseline (t_0_) to that of the HCs in order to evaluate whether MS patients displayed an impairment in their immune response due to previous immunomodulatory treatment(s).

**Figure 1:**
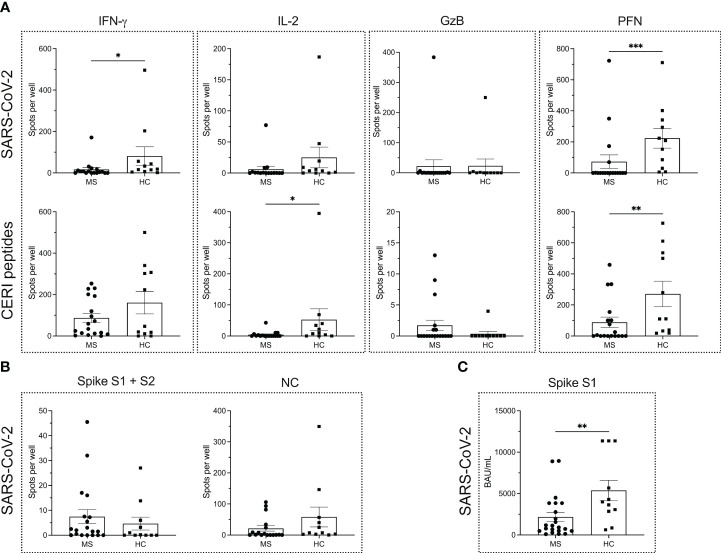
Antigen-specific immune response to SARS-CoV-2 and other viral antigens in MS patients and healthy controls. ELISPOT counts corresponding to T cell secretion of IFN-γ, IL-2, GzB and PFN in MS patients vs. HCs in response to either **(A)** SARS-CoV-2 antigens or CERI peptides. **(B)** ELISPOT counts corresponding to the B cell response against SARS-CoV-2 spike proteins S1 + S2 and NC protein in MS and HC cohorts. **(C)** Serum antibody titers against SARS-CoV-2. Mean values ± SEM are displayed in the graph. *P < 0.05; **P < 0.01; ***P < 0.001. CERI, peptide pool consisting of class I peptides from cytomegalovirus, Epstein-Barr virus, human respiratory syncytial virus, and influenza; GzB, granzyme B; HCs, healthy controls; IFN, interferon; IL, interleukin; MS, multiple sclerosis; NC, nucleocapsid; PFN, perforin.

To this end, T cell ELISPOT assays for the detection of IFN-γ, IL-2, GzB and PFN were performed, showing significantly lower levels of IFN-γ and PFN secretion in MS patients (*P* = 0.0119 for IFN-γ; *P* = 0.0005 for PFN) (**Figure 1A**) compared to HCs. On the contrary, IL-2 and GzB responses were comparable (*P* = 0.0930 for IL-2; *P* = 0.9647 for GzB) (**Figure 1A**) between the two groups. Although IL-2 secretion in general was rather low, a trend for reduced IL-2 secretion in MS patients was observed. Meanwhile, there were very few GzB responders in both cohorts. In most cases, the GzB response was either barely or not at all detectable.

To determine whether the differences in cytokine secretion pertained only to the SARS-CoV-2-specific antigens or were a more global phenomenon, we also measured the PHA- and CERI-specific T cell response. PHA was used as positive control for CD4^+^ T cell-derived responses in IFN-γ and IL-2 assays, while CERI contained MHC class-I restricted peptides and hence was used for CD8^+^ T cells in IFN-γ, IL-2, GzB and PFN assays. Since most PHA-induced responses were TNTC in both MS patients and HCs, no reliable statistics could be performed. For CERI-specific immune responses, a significantly reduced secretion of IL-2 and PFN was observed in patients with MS (*P* = 0.0105 for IL-2; *P* = 0.0077 for PFN). There were no significant differences in the secretion of either IFN-γ (*P* = 0.4921) or GzB (*P* = 0.1922) in the MS cohort compared to healthy donors (**Figure 1A**).

Furthermore, no differences were observed in the B cell response to the SARS-CoV-2 spike S1 + S2 proteins and NC protein between the MS and HC cohort (*P* = 0.4013 for the spike proteins; *P* = 0.1404 for NC protein) as shown in **Figure 1B**. However, MS patients harbored significantly lower spike S1-specific serum antibody titers (*P* = 0.0083) in comparison to HCs (**Figure 1C**).

### 3.2 Ocrelizumab treatment leads to a significant decrease in the number of SARS-CoV-2 specific peripheral B cells in MS patients

**Figure 2:**
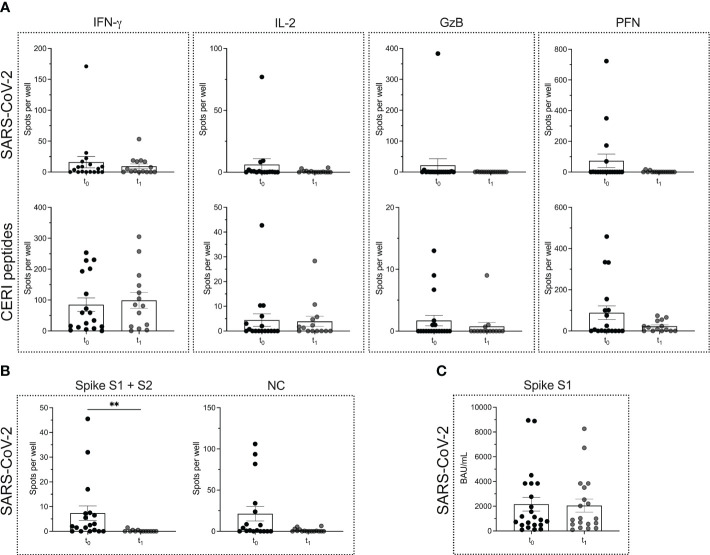
SARS-CoV-2 specific T cell and B cell responses in MS patients before and after the start of ocrelizumab therapy. ELISPOT counts corresponding to T cell secretion of IFN-γ, IL-2, GzB and PFN in MS patients before (t_0_) and after (t_1_) the start of ocrelizumab therapy in response to either **(A)** SARS-CoV-2 antigens or CERI peptides. **(B)** ELISPOT counts corresponding to the B cell response against SARS-CoV-2 spike proteins S1 + S2 and NC protein comparing the time points t_0_ *vs.* t_1_ in the MS cohort. **(C)** Serum antibody titers against SARS-CoV-2. Mean values ± SEM are displayed in the graph. **P < 0.01. CERI, peptide pool consisting of class I peptides from cytomegalovirus, Epstein-Barr virus, human respiratory syncytial virus, and influenza; GzB, granzyme B; HCs, healthy controls; IFN, interferon; IL, interleukin; MS, multiple sclerosis; NC, nucleocapsid; PFN, perforin.

T cell and B cell ELISPOT assays were performed to determine the SARS-CoV-2-specific adaptive immune response prior to (t_0_) and four weeks after (t_1_) the first cycle of ocrelizumab treatment in patients with MS. The data show no significant differences in the secretion of T cell cytokines when comparing the two time points (**Figure 2A**) (*P* = 0.9638 for IFN-γ; *P* = 0.3708 for IL-2; *P* = 0.2529 for GzB; *P* = 0.6811 for PFN). Similarly, the secretion of the cytokines in response to CERI antigens was not altered (**Figure 2A**) (*P* = 0.6597 for IFN-γ; *P* = 0.7387 for IL-2; *P* = 0.4489 for GzB; *P* = 0.5559 for PFN). As mentioned above, most PHA-induced responses were TNTC so that no reliable statistics could be performed. Taken together, our results suggest that anti-CD20 treatment did not affect the T cell compartment.

However, a significant reduction in the number of SARS-CoV-2-specific B cells was seen between the time point t_0_ *vs*. t_1_. Interestingly, the reduction in the B cell response was restricted mostly to the spike S1 + S2 proteins (*P* = 0.0012), while for the NC protein only a decreasing trend was observed (*P* = 0.0515) (**Figure 2B**). There was no significant reduction in spike S1-specific serum antibody titers when comparing the two time points t_0_ and t_1_ (*P* = 0.9484) (**Figure 2C**).

### 3.3 MS patients under ocrelizumab therapy display an impaired IL-2 and B cell response upon (re)infection with SARS-CoV-2

**Figure 3:**
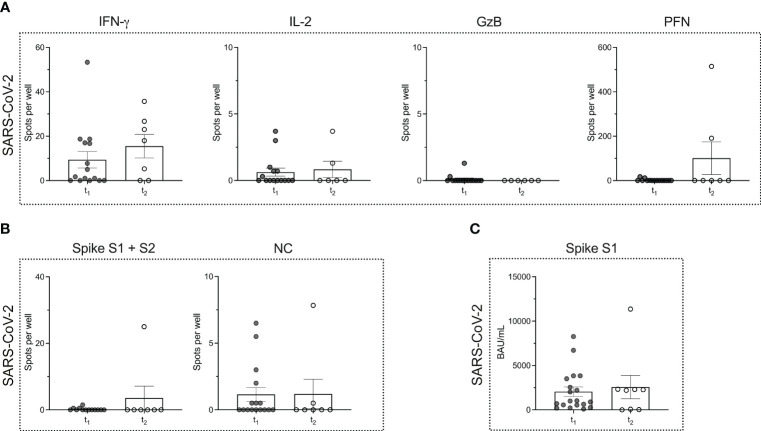
Virus-specific immune responses to infection with SARS-CoV-2 in MS patients under ocrelizumab therapy. **(A)** ELISPOT counts corresponding to SARS-CoV-2-specific T cell secretion of IFN-γ, IL-2, GzB and PFN in MS patients after (t_1_) ocrelizumab therapy and following re-infection (t_2_) with SARS-CoV-2. **(B)** ELISPOT counts corresponding to the B cell response against SARS-CoV-2 spike proteins S1 + S2 and NC protein comparing the time points t_1_ *vs.* t_2_ in the MS cohort. **(C)** Serum antibody titers against SARS-CoV-2 comparing time points t_1_ *vs.* t_2_. Mean values ± SEM are displayed in the graph. GzB, granzyme B; HCs, healthy controls; IFN, interferon; IL, interleukin; MS, multiple sclerosis; NC, nucleocapsid; PFN, perforin.

We tested *n* = 9 patients with MS, that were (re)infected with SARS-CoV-2 under ongoing ocrelizumab therapy. The time point of testing after (re)infection was termed t_2_. The mean time that elapsed between time points t_1_ and t_2_ was 226.5 ± 144.1 days. When comparing the two time points in each patient, neither a significant boost nor a decline in the immune response could be detected. Both the magnitude of the T cell (*P* = 0.2808 for IFN-γ; *P* = 0.9947 for IL-2; *P* = 0.5714 for GzB; *P* = 0.6414 PFN) and B cell responses (*P* > 0.9999 for the response to spike proteins and *P* = 0.6351 for the response to NC protein) remained comparable (**Figures 3A, B**). Moreover, there was no significant alteration in SARS-CoV-2-specific serum antibody titers (*P* = 0.7851) (**Figure 3C**).

**Figure 4:**
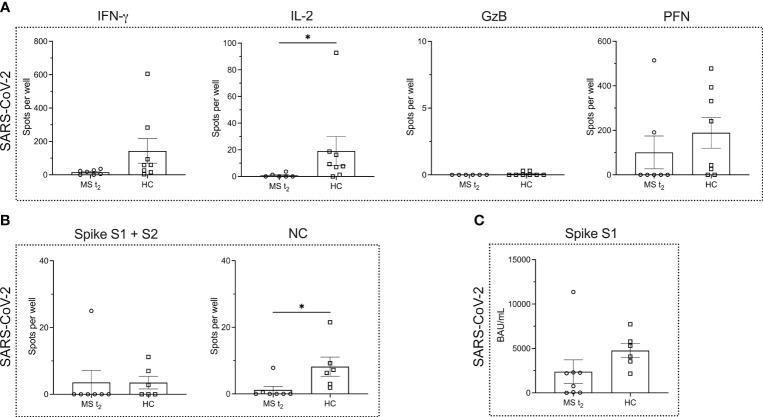
Virus-specific immune responses to infection with SARS-CoV-2 in MS patients under ocrelizumab therapy compared to infected healthy controls. **(A)** ELISPOT counts corresponding to SARS-CoV-2-specific T cell secretion of IFN-γ, IL-2, GzB and PFN in MS patients after re-infection with SARS-CoV-2 under ocrelizumab therapy (t_2_) in comparison to infected HCs. **(B)** ELISPOT counts corresponding to the B cell response against SARS-CoV-2 spike proteins S1 + S2 and NC protein comparing the time point t_2_ in the MS cohort and HCs. **(C)** Serum antibody titers against SARS-CoV-2. Mean values ± SEM are displayed in the graph. *P < 0.05. GzB, granzyme B; HCs, healthy controls; IFN, interferon; IL, interleukin; MS, multiple sclerosis; NC, nucleocapsid; PFN, perforin.

When comparing the MS (time point t_2_) and HC cohort after infection, we noted a significantly lower number of IL-2-secreting T cells in MS patients (*P* = 0.0130) (**Figure 4A**). However, for the other cytokines, the number of cytokine producing T cells remained comparable (**Figure 4A**).

While the B cell response to NC protein was significantly impaired in the MS cohort (*P* = 0.0111), the B cell response to SARS-CoV-2 spike proteins was very low in the HC cohort so that no statistical significance was reached when comparing HCs and B cell-depleted MS patients (*P* = 0.4126) (**Figure 4B**). Spike S1-specific serum IgG titers showed a decline in the MS cohort, which was, however, not statistically significant (*P* = 0.0593) (**Figure 4C**).

### 3.4 Kaplan-Meier survival analysis reveals a superior role for spike S1-specific serum antibody titers in conveying protection against SARS-CoV-2 (re)infection

Finally, we asked the question whether any of the cytokines analyzed in this study and/or the B cell or antibody response had a superior role in mediating protection against SARS-CoV-2 infection. To this end, we performed Kaplan-Meier survival analysis for the different parameters that were measured. For each parameter a cut-off was set to distinguish a low/absent response from a high response. For IFN-γ this cut-off was a mean spot count/well of 10, and for PFN a mean spot count/well of 100 was chosen. Due to the overall low IL-2 and GzB response, both cytokines were not suitable for survival analysis. With reference to the B cell compartment, a mean response < 5 spots per well was considered as low for both antigens tested, i.e., the spike S1 + S2 proteins and NC protein. Following the cut-off suggested by Schiavetti et al. (33), SARS-CoV-2 specific serum antibody titers < 659 BAU/mL were defined as low. Since the focus of this analysis was not on the comparison between MS and HCs but rather on the different immune parameters, the data of both cohorts, i.e., MS patients at baseline (t_0_) and HCs, were pooled.

**Figure 5:**
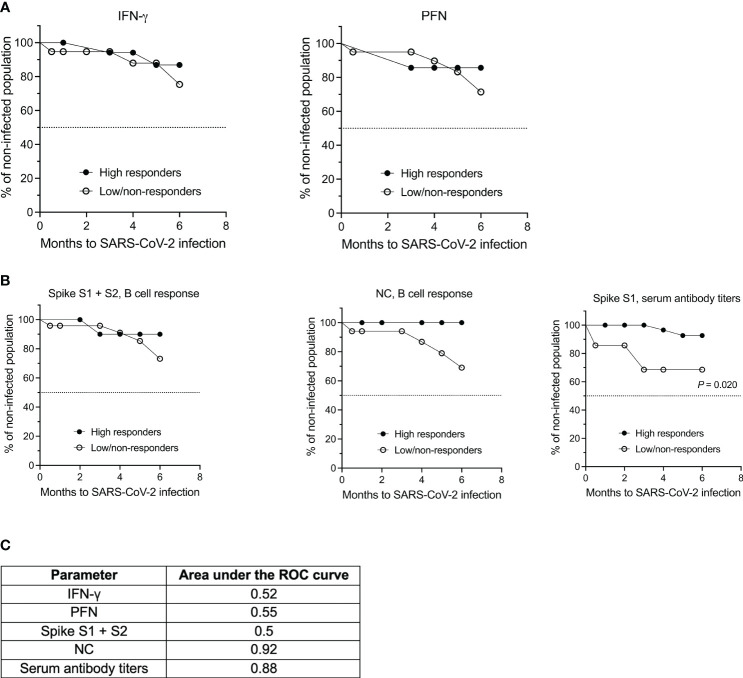
Kaplan-Meier survival and ROC analyses of both the MS and HC cohort based on the T cell, B cell and antibody response against SARS-CoV-2. **(A)** Analysis of SARS-CoV-2-specific IFN-γ and PFN secretion by T cells and **(B)** of the B cell and antibody response against spike S1 + S2 and NC antigen. **(C)** Summary of the receiver operating characteristics (ROC) analysis for the individual parameters. IFN, interferon; NC, nucleocapsid; PFN, perforin.

As shown in **Figures 5A, B**, survival analysis revealed no significant differences between non-/low responders and high responders for IFN-γ, PFN and the spike and NC protein-specific B cell response for a follow-up period of six months (*P* = 0.6332 for IFN-γ, non-/low responders: 75.4% infection-free, high responders: 86.9% infection-free; *P* = 0.6672 for PFN, non-/low responders: 71.4% infection-free, high responders: 85.7% infection-free; *P* = 0.4829 for the spike protein-specific B cell response, non-/low responders: 73.2% infection-free, high responders: 90.0% infection-free; *P* = 0.0504 for the NC-specific B cell response, non-/low responders: 69.1% infection-free, high responders: 100% infection-free). There was also no significant difference between the two groups when determining the hazard ratio for each of these parameters. However, a significant difference between the non-/low and high responder group was observed for spike S1-specific serum antibody titers (*P* = 0.0203, non-/low responders: 68.6% infection-free, high responders: 92.7% infection-free). The hazard ratio for this parameter was 7.160 [95% CI 0.3606; 142.2] for non-/low responders and 0.1397 [95% CI 0.007033; 2.773] for high responders. ROC analysis was performed to further evaluate the diagnostic performance of each classifier (**Figure 5C**). The value for the area under the ROC curve for spike S1-specific serum antibody titers was 0.88, which suggests an excellent discriminatory ability of this parameter.

## 4 Discussion

The prompt development of mRNA vaccines as an approach to tackle SARS-CoV-2 led researchers to study and understand their effectiveness especially in immunocompromised individuals or those under immunomodulatory treatments. Along these lines, one of the major questions that needed to be addressed was the safety and effectiveness of these novel mRNA vaccines in MS patients under different disease-modifying therapies (34–36). For example, studies were conducted to assess the effect of ocrelizumab, an anti-CD20 depleting monoclonal antibody, on the SARS-CoV-2-specific immune response. Since the drug leads to a highly efficient depletion of peripheral circulating B cells (37), an association between this depletion and an incomplete adaptive immune response towards SARS-CoV-2 was anticipated (38–41). Indeed, on the one hand, research showed that the SARS-CoV-2-specific B cell response was attenuated in ocrelizumab-treated MS patients (42, 43). However, on the other hand, it was suggested that anti-CD20 antibody-treated patients exhibited robust CD8^+^ T cell induction and intact CD4^+^ T cell priming (44). It was also reported that MS patients produced similar levels of IL-2 and IFN-γ, cytokines released by activated T cells, in comparison to healthy donors (45–48). Taken together, these studies provided important clues on the nature of the SARS-CoV-2-induced immune landscape in anti-CD20 treated MS patients (49).

Despite the reasonably large volume of available literature, only two studies recruited SARS-CoV-2-vaccinated MS patients before the initiation of anti-CD20 treatment. While one study measured SARS-CoV-2 spike protein-specific IgG titers in the serum of *n* = 4 MS patients prior to and after the initiation of ocrelizumab therapy (28), the second study assessed specific IgG titers in the serum as well as the spike antigen-specific CD4^+^ and CD8^+^ T cell responses in *n* = 7 patients (50). Both studies demonstrated a preservation of SARS-CoV-2 spike protein-specific IgG titers in the serum. T cell responses were, however, lower in MS patients that were vaccinated under ongoing ofatumumab therapy, another anti-CD20 monoclonal IgG antibody, compared to those that were vaccinated prior to the start of treatment (50).

The data presented here extend our current knowledge by providing a more thorough analysis of SARS-CoV-2 specific T cell and B cell responses prior to and after initiation of ocrelizumab therapy in a cohort of *n* = 23 RRMS patients. We used T cell and B cell ELISPOT assays as a quantitative method for measuring cytokine secretion and antibody production over a time period of six months, allowing longitudinal comparison of the adaptive immune response against SARS-CoV-2. Furthermore, we used CERI, a MHC class I-restricted mix of 8-11 amino acid long peptides from CMV, EBV, hRSV and influenza to monitor antigen-specific CD8^+^ T cell immunity (51). IL-2 (26), IFN-γ, GzB and PFN (29) were chosen as signature cytokines for the T cell ELISPOT to reliably detect both CD4^+^ and CD8^+^ T cell immunity against SARS-CoV-2 antigens (52). B cell ELISPOT assays were performed to determine the pathogen-specific B cell-mediated antibody response elicited by SARS-CoV-2 spike- and NC proteins (53–55). Finally, spike S1-specific serum IgG titers corresponding to the humoral immune response against the virus were determined.

We used ELISPOT for the following reasons: (*i*) ELISPOT assays assess the function of antigen-specific lymphocytes by measuring the secretion of cytokines or antibodies making them both quantitative and qualitative in nature (25–27). (*ii*) ELISPOT assays are relatively easy to perform due to the availability of commercially available kits and established protocols. (*iii*) ELISPOT assays can be standardized across multiple laboratories using common standard operating procedures making them a preferred tool for immune monitoring (and in clinical studies). (*iv*) Although there are reports of studying the frequency of antigen-specific T cells following infection or vaccination using flow cytometry (56), flow cytometry is not the method of choice for detecting antigen-specific lymphocytes. Moreover, the use of multi-color flow cytometry for monitoring the immune status of patients in a relatively small sample volume can be challenging (57).

In the first set of experiments, we compared the T cell and B cell responses at baseline (t_0_) between MS patients and HCs. As shown in **Figure 1**, IFN-γ and GzB production was comparable, but CD8^+^ T cells from MS patients produced significantly lower amounts of IL-2 (*P* = 0.0105) and PFN (*P* = 0.0077) in comparison to HCs upon stimulation with CERI peptides. Furthermore, the number of IFN-γ- and PFN-producing SARS-CoV-2-specific T cells was significantly lower in the MS cohort compared to HCs (*P* = 0.0119 and *P* = 0.0005, respectively). DMTs generally exert their therapeutic effect by downregulating cytotoxic T cell function (58) which would explain why the cellular immune response to viral antigens is impaired in the MS cohort, where most patients were already under immunomodulatory therapy before being recruited into the study (**Table 1**).

While the memory B cell response to SARS-CoV-2 spike and NC proteins was comparable between the two cohorts, the spike S1-specific serum IgG titers were significantly downregulated in MS patients (*P* = 0.0083). Therefore, on the one hand, MS patients generate a detectable SARS-CoV-2 antigen-specific B cell response but on the other hand, have significantly impaired humoral and cell-mediated immunity which, taken together, validates an overall skewed and dampened adaptive immune response in these patients under DMTs (13, 45, 59–61).

In line with previous findings (45, 62–65), we were able to confirm that CD8^+^ and CD4^+^ T cell responses to SARS-CoV-2 are retained in MS patients under anti-CD20 depletion therapy, i.e., when comparing time points t_0_ vs. t_1_, where t_0_ and t_1_ correspond to before and after the first dose ocrelizumab. Not surprisingly, however, the SARS-CoV-2 spike protein-specific memory B cell compartment was affected following ocrelizumab treatment with a significant decrease in the number of spots per well between time points t_0_ vs. t_1_ (*P* = 0.0012). Given the high peptide sequence homology between the NC protein of SARS-CoV-2 and NC proteins of other members of the coronavirus family [for example, the NC protein similarity between SARS-CoV-2 and SARS CoV-1 is 90% (66, 67)] as well as other virus groups, it has been suggested that the B cell response to the spike proteins might be more specific (68–70). Thus, our observation that the B cell response against the spike proteins S1 + S2 was significantly reduced, as opposed to the response to the NC protein, is indicative of an overall highly SARS-CoV-2-specific decrease in the B cell compartment.

It can be assumed that robust and protective B cell immunity is unlikely to contribute to the protection against COVID-19 in MS patients under long-term anti-CD20 therapy. The data presented here are in line with the publication of Alfonso-Dunn et al. where the authors concluded that the generation of a partial adaptive immune response to SARS-CoV-2 vaccination in MS patients under B cell depleting therapy is primarily driven by T cells (71). However, given that cognate interactions between B cells and T cells form the central tenet of protective immunity (72), it is important to speculate that the effect of depleting CD20^+^ B cells in MS patients would also impair, for example, B cell-dependent T cell differentiation and development of (long-lived) immunological memory (53, 73, 74). Therefore, follow-up studies need to verify whether the conserved T cell response observed between t_0_ and t_1_ is persistent beyond six months and whether this is sufficient to mount a recall response to emerging variant strains in MS patients under ocrelizumab therapy.

The effectiveness of ocrelizumab therapy in RRMS has been attributed to the depletion of CD20^+^ T cells (75). Therefore, on the one hand, it may not be surprising that patients taking ocrelizumab display impaired antigen-specific T cell responses, where a significantly lower amount of IL-2 production by T cells in the MS cohort was observed (*P* = 0.013) when comparing patients that were infected with SARS-CoV-2 under ocrelizumab therapy (time point t_2_) with infected HCs. This, on the other hand, does not support that CD8^+^ T cell immunity is compromised by anti-CD20 treatment. However, as mentioned above, no significant difference in the T cell compartment was seen either between time points t_0_ and t_1_ or t_1_ and t_2_ in the MS cohort.

Taken together, our data indicate that MS patients exhibit an overall diminished SARS-CoV-2-specific T cell response and weakened cellular immunity both before and after anti-CD20 depletion. Yet the cytokine profile of the T cells mostly remained unchanged between the time points t_0_ *vs.* t_1_ *vs.* t_2_. Although CD20^+^ T cells are a unique and transcriptionally-distinct T cell subset (76) that are known to express an increased amount of pro-inflammatory cytokines like IL-2 and granulocyte-macrophage colony-stimulating factor (GM-CSF) (75, 77) compared to CD20^-^ T cells, they represent only a small fraction of the total IFN-γ/IL-2/GzB/PFN secreting T cell population (78–80). This would explain why we did not observe any statistically significant difference in the cytokine profile of T cells in the MS cohort before and after ocrelizumab therapy. It would, thus, be interesting to quantify the presence of CD20^+^ *vs*. CD20^-^ T cells relative to the expression of the different cytokines detected by the ELISPOT assays. Of note, although the secretion of IFN-γ/IL-2/GzB or PFN by cells other than T cells cannot be excluded, their impact on the results as shown in this study should be marginal since anti-CD28 was added in the ELISPOT assay and spot morphology specifically indicated a T cell-derived response (81).

Finally, Kaplan-Meier survival analysis suggested that spike S1-specific serum antibody titers may be a key parameter to predict the probability of (re)infection. Hence, monitoring this response longitudinally might be a reasonable approach to predict the risk of SARS-CoV-2 (re)infection in patients or healthy donors, in particular if they belong to a high-risk group of developing severe side effects of COVID-19. In this case, we pooled both cohorts (i.e., HCs and MS patients) into a single group to assess the chance of (re)infection with SARS-CoV-2 in any given individual, independent of their health status (i.e., patients *vs.* corresponding controls), but rather based on their T cell cytokine and/or B cell or antibody responses analyzed in this study.

To summarize, we were able to confirm that ocrelizumab treatment triggered a significant reduction of SARS-CoV-2-specific circulating B cells in RRMS patients. Our data imply that infection with SARS-CoV-2 does not induce a significant boost in the antigen-specific T cell and B cell responses in patients under ongoing ocrelizumab therapy. Furthermore, considering that the secretion of cytokines in response to SARS-CoV-2 was significantly altered after infection, our findings suggest a possibly skewed immune response to viral antigens under ocrelizumab treatment, given that cytotoxic T cells play a critical role in anti-viral defenses (54, 55). Yet, infections with viruses other than SARS-CoV-2 were not assessed in this study, so that there is a potential source of error as T cell reactivity against other viruses such as influenza might still be enhanced after infection despite treatment.

A weakness of our study is that we did not discriminate between CD4^+^ and CD8^+^ cells in our T cell ELISPOT assays. Additionally, the PepTivator^®^ SARS-CoV-2 antigen pool contained both MHC class I- and II-restricted peptides. Hence, the effect of CD20-depleting therapy on the individual CD4^+^ *vs.* CD8^+^ T cell-derived responses to SARS-CoV-2 cannot be confirmed but only assumed based on the cytokine signature of a particular T cell subset. For example, GzB and PFN are more likely to indicate a CD8^+^ T cell response (82–84). Furthermore, given the access to only a limited volume of patient material (as approved by the ethics committee), we were not able to precisely assess the extent to which T cell and/or innate immune responses compensate for the attenuated B cell and humoral responses in conferring protection in MS patients under CD20 depleting therapy (58).

One strength of the study was the experimental set-up with baseline samples taken before initiation of B cell depletion with ocrelizumab. However, it should be noted that only a few of these patients were treatment-naïve, and most had received immunosuppressant or immunomodulating therapy before blood samples were drawn at t_0_. Finally, another strength of this study was the combination of the SARS-CoV-2 IgG II Quant and B cell ELISPOT assay, which allowed us to distinguish between SARS-CoV-2-specific serum antibody titers and memory B cells.

## 5 Conclusion

An established immune memory is essential in the defense against SARS-CoV-2 infection. The current manuscript highlights the effect of ocrelizumab, an anti-CD20 depleting monoclonal antibody, on the immune response against SARS-CoV-2 in patients with MS. Using a broad immune monitoring approach, the longitudinal measurement of patient samples presented in this study provides useful information on the persistence of a SARS-CoV-2-specific adaptive immune response over the course of ocrelizumab therapy. Our data may pave the way to determine ideal vaccination intervals in individual patients and to foster a better understanding of the parameters that provide long-term protection against severe COVID-19. Future studies should be carried out using multiomics approaches or multi-color flow cytometry with larger cohort sizes to validate the findings presented here.

## Data availability statement

The original contributions presented in the study are included in the article/supplementary material. Further inquiries can be directed to the corresponding author.

## Ethics statement

The studies involving humans were approved by Ethics Committee of the Medical Faculty, University of Bonn. The studies were conducted in accordance with the local legislation and institutional requirements. The participants provided their written informed consent to participate in this study.

## Author contributions

EG: Conceptualization, Formal Analysis, Investigation, Methodology, Visualization, Writing – original draft. AW: Investigation, Writing – review & editing. MV: Project administration, Resources, Writing – review & editing. TH: Conceptualization, Project administration, Resources, Supervision, Writing – review & editing. RC: Formal Analysis, Investigation, Methodology, Supervision, Validation, Visualization, Writing – original draft, Writing - review & editing. SK: Conceptualization, Formal Analysis, Funding acquisition, Investigation, Methodology, Project administration, Resources, Supervision, Validation, Writing – original draft, Writing - review & editing.
